# Combined Effects of Three High-Energy Charged Particle Beams Important for Space Flight on Brain, Behavioral and Cognitive Endpoints in B6D2F1 Female and Male Mice

**DOI:** 10.3389/fphys.2019.00179

**Published:** 2019-03-12

**Authors:** Jacob Raber, Joy Yamazaki, Eileen Ruth S. Torres, Nicole Kirchoff, Keaton Stagaman, Thomas Sharpton, Mitchell S. Turker, Amy Kronenberg

**Affiliations:** ^1^ Department of Behavioral Neuroscience, Oregon Health & Science University, Portland, OR, United States; ^2^ Department of Neurology, Division of Neuroscience ONPRC, Oregon Health & Science University, Portland, OR, United States; ^3^ Department of Radiation Medicine, Division of Neuroscience ONPRC, Oregon Health & Science University, Portland, OR, United States; ^4^ Department of Microbiology, Oregon State University, Corvallis, OR, United States; ^5^ Department of Statistics, Oregon State University, Corvallis, OR, United States; ^6^ Department of Molecular and Medical Genetics, Oregon Health & Science University, Portland, OR, United States; ^7^ Oregon Institute of Occupational Health Sciences, Oregon Health & Science University, Portland, OR, United States; ^8^ Biological Systems and Engineering Division, Lawrence Berkeley National Laboratory, Berkeley, CA, United States

**Keywords:** object recognition, home cage activity, depressive-like behavior, CD68, BDNF, gut microbiome, charged particle radiation, space flight

## Abstract

The radiation environment in deep space includes the galactic cosmic radiation with different proportions of all naturally occurring ions from protons to uranium. Most experimental animal studies for assessing the biological effects of charged particles have involved acute dose delivery for single ions and/or fractionated exposure protocols. Here, we assessed the behavioral and cognitive performance of female and male C57BL/6J × DBA2/J F1 (B6D2F1) mice 2 months following rapidly delivered, sequential irradiation with protons (1 GeV, 60%), ^16^O (250 MeV/n, 20%), and ^28^Si (263 MeV/n, 20%) at 0, 25, 50, or 200 cGy at 4–6 months of age. Cortical BDNF, CD68, and MAP-2 levels were analyzed 3 months after irradiation or sham irradiation. During the dark period, male mice irradiated with 50 cGy showed higher activity levels in the home cage than sham-irradiated mice. Mice irradiated with 50 cGy also showed increased depressive behavior in the forced swim test. When cognitive performance was assessed, sham-irradiated mice of both sexes and mice irradiated with 25 cGy showed normal responses to object recognition and novel object exploration. However, object recognition was impaired in female and male mice irradiated with 50 or 200 cGy. For cortical levels of the neurotrophic factor BDNF and the marker of microglial activation CD68, there were sex × radiation interactions. In females, but not males, there were increased CD68 levels following irradiation. In males, but not females, there were reduced BDNF levels following irradiation. A significant positive correlation between BDNF and CD68 levels was observed, suggesting a role for activated microglia in the alterations in BDNF levels. Finally, sequential beam irradiation impacted the diversity and composition of the gut microbiome. These included dose-dependent impacts and alterations to the relative abundance of several gut genera, such as *Butyricicoccus* and *Lachnospiraceae.* Thus, exposure to rapidly delivered sequential proton, ^16^O ion, and ^28^Si ion irradiation significantly affects behavioral and cognitive performance, cortical levels of CD68 and BDNF in a sex-dependent fashion, and the gut microbiome.

## Introduction

A unique feature of the space radiation environment is the presence of galactic cosmic rays (GCR) and solar particle events (SPE). The former involves fully ionized atomic nuclei for all naturally occurring elements from hydrogen to uranium, while the latter includes predominantly low- to medium-energy protons with a small heavy ion component. Space radiation may pose a hazard to space flight crews during the mission. Prior NASA-supported studies have typically involved single-particle exposures with protons (Clapp et al., 1974; Rabin et al., 1991; Chang et al., 2005; Bellone et al., 2014; Sweet et al., 2014; Parihar et al., 2015; Sokolova et al., 2015; Impey et al., 2016a,b; Rudobeck et al., 2017), ^16^O (Poulose et al., 2011; Sweet et al., 2014; Rabin et al., 2015; Raber et al., 2015a,b,c), ^56^Fe (Shukitt-Hale et al., 2003; Rola and Fike, 2004; Villasana et al., 2008; Vlkolinsky et al., 2008; Cherry, 2012), ^28^Si (Bellone et al., 2014; Raber et al., 2014a,b; Raber et al., 2015a,b,c; Whoolery et al., 2017), or combined effects of two-particle exposures, involving for example protons (150 MeV/n, 0.1 Gy) and ^56^Fe ions (600 MeV/n, 0.5 Gy) (Raber et al., 2015a,b,c), on hippocampal function. Other studies have considered executive function (Britten et al., 2014; Davis et al., 2015; Britten et al., 2017). However, there is a clear gap in our knowledge regarding exposure to complex radiation fields involving more than two particles, a scenario very pertinent to exposures of astronauts during space missions.

The environmental conditions astronauts experience during space missions, especially long space missions, include not only ionizing radiation, but also psychological and physical stressors (Stuster, 2016). Exposure to space radiation might cause not only cognitive (Cucinotta et al., 2014) but also behavioral alterations and modulate the individual response of astronauts to psychological and physical stressors. Therefore, evaluating behavioral performance, including the response to controlled environmental emotional stressors, is also important. Behavioral measures pertinent to the successful performance of the astronauts during space missions include measures of anxiety, depression, and circadian activity (Stuster, 2016).

With regard to cognitive performance, object recognition (Rabin et al., 2008; Raber et al., 2015a,b,c; Impey et al., 2016a,b) and contextual and cued fear learning and memory (Villasana et al., 2010a,b; Raber et al., 2013a,b; Raber et al., 2014a,b; Raber et al., 2015a,b,c; Whoolery et al., 2017) were sensitive to detect effects of certain high-energy charged particle exposures of relevance to space flight using a ground-based accelerator as the source of these ions. Some types of accelerator-derived charged particles also affected response to a novel environment. Baseline activity in a novel environment was reduced in 5-month-old C57BL/6J mice 3 months following ^56^Fe ion irradiation (600 MeV/n, 174 keV/μm, 0.5 Gy) (Allen et al., 2015). While the object recognition and fear conditioning tests detected effects of charged particle exposures, the relationship between the dose of each ion, its linear energy transfer (LET—a measure of the density of energy deposited in a local area), and cognitive performance on these tests is complex (Rabin et al., 2014; Raber et al., 2015a,b,c). As most mouse radiation studies have been performed using C57Bl6/J mice and genetic factors are anticipated to modulate the radiation response, it is important to assess radiation effects in other genetic backgrounds, such as the B6D2F1 background (Raber et al., 2015a,b,c; Raber et al., 2016) used in the current study.

The search for basic mechanisms underlying the effects of charged particle radiation on behavior and cognitive performance has included examination of the levels of key signaling molecules. As an example, inhibited transcription of brain-derived neurotrophic factor (BDNF) has been implicated after clinically relevant radiation doses to the whole brain that produced cognitive injury in rats (30 Gy; 4 MV electrons) and mice (10 Gy; 6 MV photons) (Ji et al., 2014; Son et al., 2015). The effect of microglia, resident macrophages in the brain, on learning-dependent synapse formation involves the release of BDNF (Parkhurst et al., 2013). This release is important for cognitive performance (Zafra et al., 1990; Cheng and Mattson, 1994; Ghosh et al., 1994; Hellweg and Jockers-ScherÅbl, 1994; Lo, 1995; Kang et al., 1996; Linnarsson et al., 1997; Wardle and Poo, 2003; Piepmeier and Etnier, 2015). Activation of microglia, important in neuroinflammation, triggers the release of BDNF, which in turn induces the proliferation and prolonged activation of microglia (Gomes et al., 2013; Mizobuchi et al., 2014; Zhang et al., 2014). CD68 (macrosialin), a lysosome-associated membrane glycoprotein, is a marker of activated microglia (Neuen-Jacob et al., 1993; Tanaka et al., 2013) and CD68 levels were increased in the mouse brain following a moderate (2 Gy) whole-body exposure to gamma rays (Allen et al., 2014; Acharya and Patel, 2015). An increase in activated microglia, assessed as immunoreactive ED-1, cells was also reported in the medial prefrontal cortex of male transgenic mice, Thy1-EGFP MJrsJ mice, 15 and 20 weeks following ^16^O (600 MeV/n; 5 or 30 cGy) and ^48^Ti (600 MeV/n; 5 or 30 cGy) ion irradiation at 6 months of age (Parihar, 2016).

Microtubule-associated protein 2 (MAP-2) is a dendritic protein important for stabilizing microtubuli and dendritic plasticity (Johnson and Jope, 1992; Matesic and Lin, 1994). MAP-2 is required for dendrite elongation (Harada et al., 2002). MAP-2 was shown to be a sensitive marker for age-related changes in rodents (Benice et al., 2006; Peister et al., 2006) and nonhuman primates (Haley et al., 2010) and can also be affected by irradiation. When 1-month-old C57BL/6J mice were trained for contextual fear conditioning, followed by irradiation 1 day later (X-rays, whole body, 4 Gy) and extinction was assessed over 8 days starting 2 weeks after training, MAP-2 levels in the hippocampus were increased in mice that received five shocks during training (Olsen et al., 2014). This increase might be due to disinhibition of GABA-ergic neurotransmission (Kugelman et al., 2016). In mice expressing human apolipoprotein E3 under control of the mouse apoE promoter, MAP-2 immunoreactivity in the hippocampus, cortex, and amygdala was increased 3 months following ^137^Cs irradiation (head only, 10 Gy) at 2 months of age (Villasana et al., 2010a,b).

In the present study, we tested the hypotheses that sequential beam irradiation would affect behavioral and cognitive performance and that these effects would be associated with alterations in cortical levels of BDNF, MAP-2, and CD-68 and/or alterations in the gut microbiome. We assessed the effects of three rapidly delivered sequential ion beams including sparsely ionizing protons and two more densely ionizing heavy ions on behavior and cognitive performance of female and male mice and queried whether those effects are associated with alterations in cortical BDNF, MAP-2, and CD68 levels. One way space radiation might affect the brain is through alteration in the diversity of the gut microbiome and gut-liver-brain axis (Tilg and Kaser, 2011; Foster and McVey Neufeld, 2013; Ohland et al., 2013; Ghaisas et al., 2016; Allen, 2017; Turroni et al., 2017). ^16^O ion irradiation (600 MeV/n, 0.1 and 0.25 Gy) was shown to reduce alpha-diversity, defined as the diversity within the sample, in C57BL/6J male mice 10 and 30 days after exposure at 6 months of age (Casero et al., 2017), a 13-day space flight affected the gut microbiome in C57BL/6J female mice (Ritchie, 2015) (for a review, please see (Cervantes and Hong, 2016)), and it is important to monitor the temporal dynamics of microbiota components in people sharing a confined environment during space missions (Turroni et al., 2017). Recently, we reported that the environmental toxin pertinent to Parkinson’s disease, 1-methyl-4-phenyl-1,2,3,6-tetrahydropyridine (MPTP), affected the diversity of the gut microbiome and that there were significant associations between microbiome alpha-diversity and sensorimotor performance, as well as microbiome composition and fear learning (Torres et al., 2018). Therefore, we also assessed the effects of sequential beam exposure to a simulated deep space radiation environment on the gut microbiome of female and male B6D2F1 mice.

## Methods and Materials

### Animals, Radiation Exposure, Study Design, and Home Cage Monitoring

The experimental B6D2F1 mice were bred in Dr. Turker’s laboratory at Oregon Health & Science University (OHSU). Breeding cages contained two female mice and one male mouse and the husbandry procedures were consistent with Jax Labs recommendations. Mice were transported by commercial air carrier, approximately 1 week prior to irradiation, from OHSU to Brookhaven National Laboratory (BNL). They were irradiated with a series of three charged particle beams, or sham-irradiated, at 4–6 months of age at the NASA Space Radiation Laboratory (NSRL) at BNL. The doses for this study were specified by the NASA Space Radiation Health Program Manager, with the lower doses being especially germane for long-duration missions with the highest dose included as a benchmark (Zeitlin, et al., Science, 2013). For this study with three ion beams, the goal was to have a substantial exposure from protons, the most abundant ion species in the GCR, a significant dose from a representative ion with Z between 3 and 9, and a significant dose from a representative ion with Z greater than 10. Beams were chosen based on prior work with individual ions with this B6D2F1 mouse model (Raber et al., 2015a,b,c). The following exposures were given in rapid sequence to each exposed mouse, with three mice irradiated at the same time: 60% of the total dose delivered with 1 GeV protons (LET = 0.24 keV/μm), 20% of the total dose delivered with 250 MeV/n ^16^O ions, (LET = 25 keV/μm), and 20% of the total dose delivered with 263 MeV/n ^28^Si ions (78 keV/μm). Mice were given whole-body exposure in the absence of anesthesia and were exposed with their flanks horizontal to the beam and were irradiated in small Plexiglas boxes drilled with air holes according to our standard methods (Kronenberg et al., 2009). Dosimetry was performed with a series of parallel plate ionization chambers calibrated with a NIST-traceable thimble ionization chamber (EG&G) according to our standard methods (Kronenberg et al., 2009). The breakdown of the number of mice per dose and sex was: *N* = 86 mice; 39 males (sham irradiation: *n* = 9 mice, 25 cGy: *n* = 12 mice; 50 cGy: *n* = 11 mice; 200 cGy: *n* = 6 mice) and 48 females (sham irradiation: *n* = 12 mice; 25 cGy: *n* = 12 mice; 50 cGy: *n* = 12 mice; 200 cGy: *n* = 12 mice). Based on the increase in the percentage of female astronauts participating in space missions, potential sex differences in susceptibility of the brain to develop behavioral alterations and cognitive injury following exposure to space radiation, and potential sex differences in the response of particular biomarkers in the brain to space irradiation, it is important to consider including both sexes in the experimental design.

Mice were shipped back to OHSU approximately 1 week following irradiation and tested for behavioral and cognitive performance 2 months later. Mice were group housed at OHSU with three mice per cage on a ventilated Thoren rack throughout the experimental period, except during week 1 only when a subset of 24 male mice (six mice/dose from each dose group) were singly housed and home cage activity was monitored on a conventional Metro rack using an MLog (BioBServe, Germany) home cage sensor system, as described (Johnson et al., 2015). The mice housed in the same cage received the same radiation dose. For the home cage study only, female mice were not tested due to equipment limitations. All remaining tests were performed on both males and females. All mice were kept under a constant 12-h light: 12-h dark cycle, and water and food (PicoLab Rodent Diet 20, no. 5053; PMI Nutrition International, St. Louis, MO, USA) were provided *ad libitum.* Three sham-irradiated females were removed from the study for a kidney mutagenesis assay after week 2 and did not participate in fear conditioning and passive avoidance testing. All procedures were approved by the Institutional Animal Care and Use Committees at OHSU and BNL and were in compliance with all Federal regulations.

### Behavioral and Cognitive Testing

All behavioral and cognitive testing was conducted at OHSU by experimenters who were blinded to radiation dose. As indicated above, home cage activity monitoring took place during week 1. Body weights were also recorded. Starting on day 1 of week 2, exploratory activity and measures of anxiety were assessed in the open field for 3 days. The mice were tested for novel object recognition during the two subsequent days. Starting on day 1 of week 3, depressive behavior was assessed using the forced swim test. During the remainder of week 3, hippocampus-dependent contextual and hippocampus-independent cued fear learning and memory were assessed. During week 4, fear learning and memory were assessed using the passive avoidance test. The behavioral and cognitive paradigms used are described in detail below.

### Open Field and Novel Object Recognition

The open field was used to assess measures of anxiety, locomotor, and exploratory behavior. Mice were singly placed in a brightly lit (average: 400 lux) clear plexiglass arena (40.64 × 40.64 cm) (Kinder Scientific, Poway, CA, USA) for 5-minute trials, once each day for three consecutive days. Arenas were cleaned with 0.5% acetic acid between each trial. Two white noise-generating devices, (average: 85 dB, Kinder Scientific, Poway, CA, USA) were used, one on each side of the arena platforms, during the entirety of testing. Movement of the mice and durations spent in the center of the arena (center 20 cm square area) were recorded and analyzed using Ethovision XT 7 video tracking software (Noldus Information Technologies, Wageningen, the Netherlands). After habituation to the arena for 3 days, novel object recognition was assessed by placing two identical objects 10 cm apart in the arena on the fourth day, then replacing one object with a novel object on the fifth day. Trials on the fourth and fifth days were for 10 min each. Videos recorded were viewed by experimenters who hand scored durations of time spent exploring each object. Time spent exploring the novel object versus the familiar object on day 5, expressed as a percentage of the total object exploration time in the trial, was used to determine object recognition memory.

### Porsolt Forced Swim Test

The Porsolt forced swim test (Porsolt et al., 1977) was used to assess depression-like behavior. Each mouse was placed individually in a 2000-ml glass beaker (diameter = 12.7 cm) containing 1,600 ml of room temperature (21°C) water for a single 6-min trial (four glass beakers were used per trial to test four mice simultaneously). Videos were recorded and the last 5 min of each trial was hand scored for time spent immobile. Immobility was characterized by cessation of any movement other than the minimum motion needed to keep the head above water. The percentage of time spent immobile was used as a measure of depression-like behavior and/or learned helplessness.

### Fear Conditioning

Contextual and cued fear conditioning were used to assess hippocampus-dependent contextual associative memory and hippocampus-independent cued associative memory (Anagnostaras et al., 2010) using near-infrared (NIR) video and automated analysis, and Video Freeze automated scoring software (Med Associates Inc., St. Albans, VT, USA). In the fear conditioning tests, mice learned to associate an environmental context or cue (tone, conditioned stimulus, CS) with a mild foot shock (unconditioned stimulus, US). Upon re-exposure to the training context, or a new environment in which the mice are exposed to a tone that was present during training, associative learning is assessed based on freezing behavior, characterized by absence of all movement besides respiration. On the first day (training), the mice were individually placed inside a white LED-lit (100 lux) fear conditioning chamber with a metal grid floor and allowed to habituate for a 90-s baseline period. This was followed by a 80-dB, 2,800-Hz tone (conditioned stimulus (CS) or cue) lasting for 30 s and co-terminating with a 2-s, 0.7-mA foot shock (unconditioned stimulus or US) at 120 s. Five tone-shock pairings were used, with an inter-shock interval (ISI) of 90 s. Measurements of average motion (cm/s) and percentage of time freezing were analyzed during the baseline period (prior to the first tone), and during each subsequent ISI and CS (tone/cue) to assess acquisition of fear memory. Chambers were cleaned between trials with a 0.5% acetic acid solution. On day 2, the mice were placed back into the same context as used on the training day, for a single 5-min trial, and freezing behavior was measured in the absence of either tones or shocks to assess contextual associative memory. Four hours later, the mice were placed into a novel context, containing a smooth white plastic covering the wire grid floor, a “tented” black plastic ceiling, and scented with hidden vanilla extract-soaked nestlets. The chambers were cleaned between trials with a 10% isopropanol solution. Each trial consisted of a 90-s baseline, then a 180-s, 80-dB, 2,800-Hz tone and freezing behavior was analyzed as an indicator of cued associative memory.

### Passive Avoidance

Emotional learning and memory were assessed using the passive avoidance test. Mice were placed individually into a passive avoidance chamber, consisting of two sound-attenuating, equally sized compartments (24.5 cm × 19 cm × 23 cm) separated by a gate (Hamilton-Kinder, Poway, CA, USA). On the first day (training), after a 5-s habituation, a light turned on in the compartment containing the mouse. Simultaneously, the gate to the adjoining dark compartment opened. Mice preferred to cross into the dark compartment rather than remaining in brightly lit environments. After crossing over into the dark compartment, the mice received a 3-s foot shock (0.35 mA), and the mice were immediately removed from the chamber. Mice that did not cross over into the dark compartment within the 120-s training trial were gently pushed by the experimenter into the compartment and subsequently received the foot shock. Due to technical failure, five mice did not receive a shock and therefore their data were removed from the analysis. On day 2 (24-h retention period), the mice were placed back into the lit compartment, and latency to re-enter the dark compartment was measured, up to 300 s. Mice were removed immediately from the chamber after crossing over. Mice that did not cross over were removed from the chamber after 300 s. No shock was administered during the day 2 trials. On both days, chambers were cleaned between trials with 0.5% acetic acid. Latency to cross on day 2 was analyzed as a measure of fear memory.

### MAP-2, CD68, and BDNF ELISAs

For assessments of MAP-2, CD68, and BDNF levels, the mice were euthanized and cortical regions of their brains dissected. The cortex of each mouse brain was homogenized and a protein assay was performed using a BCA kit (Fisher Scientific, Chicago, IL), as described. MyBioSource (San Diego, CA) mouse BDNF (Catalog number MBS495345), CD68 (Catalog number MBS2601301), and MAP-2 (Catalog number MBS725632) ELISAs were used to determine cortical levels of BDNF, CD68, and MAP-2 according to the manufacturer’s instructions, 3 months following sham irradiation or exposure to the three rapidly switched sequential ion beams at BNL. The standard curve was run in duplicate and the samples as single samples. There were nine samples per radiation condition per brain region. Based on the optical density values, the MAP-2, CD68, and BDNF levels in the samples were calculated using GraphPad Prism software (San Diego, CA).

### Microbiome Sequencing

Fecal boli samples for each cage and each dose were collected during open field testing (2 months post-irradiation) and stored at −80°C until analyses could be performed. Bacterial 16S rDNA sequences were extracted and sequenced as previously described (Gaulke et al., 2016). Briefly, DNA was extracted from collected fecal pellets using the QIAgen DNeasy Power Soil kit (Qiagen, Hilden, Germany) following the manufacturer’s protocol with the addition of an incubation step of 10 min at 65°C before bead beating. The V4 region of the 16S rDNA gene was amplified using the Earth Microbiome Project 16S PCR protocol. PCRs were conducted in triplicate for each sample and amplicons were run on a 1% agarose gel for quality control. PCRs were cleaned with the UltraClean PCR clean-up kit (Qiagen, Hilden, Germany) and diluted to produce 200 ng of DNA per sample. The prepared libraries were submitted to the Oregon State University Center for Genome Research and Biocomputing for 250 bp paired-end sequencing on an Illumina MiSeq instrument. Quality control, exact sequence variants clustering, and chimera removal were conducted using the dada2 package (Callahan et al., 2016) for R (R Core Team, 2017). Dada2 also assigned taxonomy to the sequence variants utilizing the Silva taxonomic training data formatted for dada2. Exact sequence variants were aligned using mafft (Katoh and Standley, 2013) and a phylogenetic tree of the bacterial community from all samples was generated using FastTree (Price et al., 2010). Sequences were rarefied to 10,423 sequences per sample.

### Statistical Analyses

All data are shown as mean ± standard error of the mean (SEM). Statistical analyses were performed using SPSS™ (version 22, Chicago, IL) software packages. The data were analyzed using ANOVAs with radiation and sex as between-group factors, followed up by *post hoc* tests when appropriate. When sex was not a significant factor, it was dropped from the model. Performance over multiple trials was analyzed by repeated-measures ANOVA. If violation of sphericity occurred indicating that the variances of the differences between all combinations of the groups were not equal (Mauchly’s test), Greenhouse-Geisser corrections were used. Bonferroni’s *post hoc* and Dunnet’s tests were used. All figures were generated using GraphPad Prism software (San Diego, CA). We considered *p* < 0.05 as statistically significant. Statistical analysis of microbiome data relied on non-parametric tests of association as defined in the results. Multiple tests were corrected through quantification of the false discovery rate.

A variety of statistical tests were applied to measure the microbiome’s association with study covariates. Linear models were used to quantify the relationship between sequential three-beam irradiation dose and each sample’s Shannon entropy, which is a measure of community evenness. Two-tailed Wilcoxon tests measured whether exposure to any dose of radiation affected the Shannon entropy of the gut microbiome. Principal coordinates analyses based on the Canberra distance visualized the variation in microbiome community composition across samples. PERMANOVA tests, as implemented by the Adonis function, determined if community composition varied in association with radiation exposure. Non-parametric tests of association, specifically Kendall’s tau, quantified the covariation between the abundance of specific genera of gut microbes and radiation dose. Multiple tests were corrected through quantification of the false discovery rate (fdr) to control for type I errors.

## Results

### Body Weights and Home Cage Activity

Based on observations by the researchers and animal staff, the rapid, sequential, three beam exposures were well tolerated by the animals and no obvious adverse effects were observed during the post-irradiation follow-up and testing periods at any dose tested. Body weights recorded post-irradiation and prior to behavioral testing did reveal a pronounced effect of sex (*F* = 71.921, *p* < 0.001), as would be expected, but did not show an overall effect of radiation (*F* = 1.55, *p* = 0.208) or sex × radiation interaction (*F* = 0.858, *p* = 0.466) (not shown).

**Figure 1:**
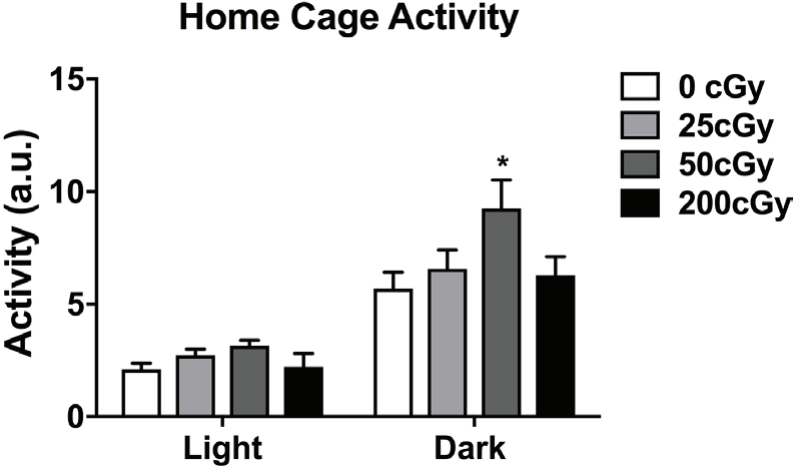
Home cage of male mice that were sham-irradiated or mixed beam-irradiated. During the dark period, mice irradiated with 50 cGy treatment displayed significantly higher activity than sham-irradiated mice (*p* = 0.036). There were no significant effects of irradiation seen in mice irradiated with 25 or 200 cGy, nor were there any significant effects of irradiation on activity levels seen during the light cycle. The light and dark periods were analyzed separately using one-way ANOVAs.

Home cage activity of mice (*n* = 24 male mice, six mice per dose) was analyzed using separate one-way ANOVAs for the light and dark periods using treatment dose as the between-subjects factor (Figure 1). During the dark period, the 50 cGy group showed higher activity levels than sham-irradiated mice (*p* = 0.036, Bonferroni’s correction for multiple comparisons and Dunnett’s *t*-test as *post hoc* test). However, there was not a significant effect of irradiation when all dose groups were considered (*F*(3,20) = 2.851; *p* = 0.063). Activity levels during the dark period in mice irradiated with 25 or 200 cGy were not different from those in sham-irradiated mice. In contrast to the dark period, there was no effect of irradiation (*F*(3,20) = 1.732; *p* = 0.193) during the light period.

### Activity Levels and Measures of Anxiety in the Open Field

**Figure 2:**
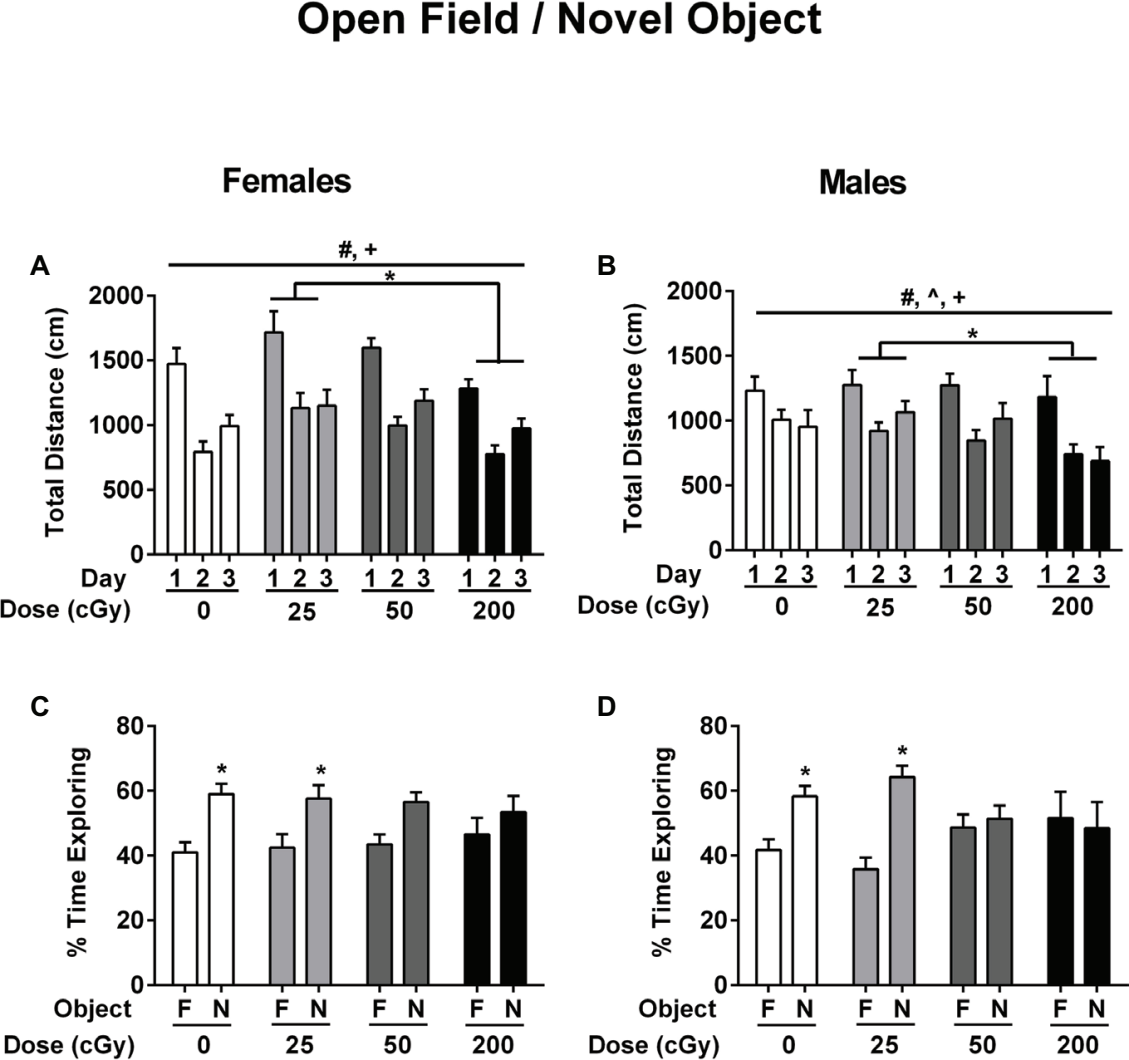
Performance of female **(A,C)** and male **(B,D)** mice that received sham irradiation or mixed beams in the open field **(A,B)** and the novel object recognition **(C,D)** tests. **(A/B)** The female and male mice habituated to the open field. The distance traveled decreased over the 3 days of exposure to the open field (Mauchly’s test of sphericity: *p* = 0.181, Greenhouse–Geisser corrected, *F*_1.917,149.509_ = 77.654; *p* < 0.001) and female mice showed higher activity levels than male mice (*F*_1,78_ = 7.387; *p* = 0.008). In addition, there was an effect of radiation on activity levels (*F*_1,78_ = 3.860; *p* = 0.012). Mice irradiated with 25 cGy showed higher activity levels than mice irradiated with 200 cGy (*p* = 0.010, *t* test). **(C/D)** Female **(C)** and male **(D)** sham-irradiated mice and those irradiated at the 25 cGy preferentially explored the novel object (females sham-irradiated mice: *p* = 0.00047; female mice irradiated with 25 cGy: *p* = 0.019; male sham-irradiated mice: *p* = 0.0023; male mice irradiated with 25 cGy: *p* < 0.0001, *t* tests) but female **(C)** and male **(D)** mice irradiated with 50 or 200 cGy were impaired and did not.

Effects of rapid, sequential three-beam irradiation on activity levels and measures of anxiety were analyzed in the open field. Total distance traveled (cm) and duration in the center (s) in the open field were analyzed using ANOVA with repeated measures for the three trials over three consecutive days. Across all female (Figure 2A) and male (Figure 2B) treatment groups, the total distance traveled decreased over the 3 days of exposure to the open field (Mauchly’s test of sphericity: *p* = 0.181, Greenhouse–Geisser corrected, *F*_1.917,149.509_ = 77.654; *p* < 0.001), indicating successful habituation to the open field. In addition, females showed higher activity levels than males overall (*F*_1,78_ = 7.387; *p* = 0.008). There was also an effect of radiation on activity levels (*F*_1,78_ = 3.860; *p* = 0.012). Mice irradiated with 25 cGy showed higher activity levels than mice irradiated with 200 cGy (*p* = 0.010, *t* test). Finally, there were significant interactions between day and sex for total activity levels (*F*_1.917,149.509_ = 4.076; *p* = 0.020) and time spent in the center of the open field. (*F*_1.86,145.047_ = 3.695; *p* = 0.030). However, there were no main effects seen for either measure.

### Novel Object Recognition

Next, the effects of rapid, sequential three-beam irradiation on novel object recognition was assessed. Female (Figure 2C) and male (Figure 2D) sham-irradiated mice and those irradiated at the lowest 25 cGy dose showed a preference for exploring the novel object (female sham-irradiated mice: *p* = 0.00047; female mice irradiated with 25 cGy: *p* = 0.019; male sham-irradiated mice: *p* = 0.0023; male mice irradiated with 25 cGy: *p* < 0.0001, *t* tests). However, female (Figure 2C) and male (Figure 2D) mice irradiated with 50 (females: *p* = 0.109; males: *p* = 0.638) or 200 cGy (females: *p* = 0.348; males: *p* = 0.786) were impaired and did not show a significant preference for exploring the novel object, in contradistinction to the results obtained at the lowest dose of 25 cGy.

### Forced Swim Test

**Figure 3:**
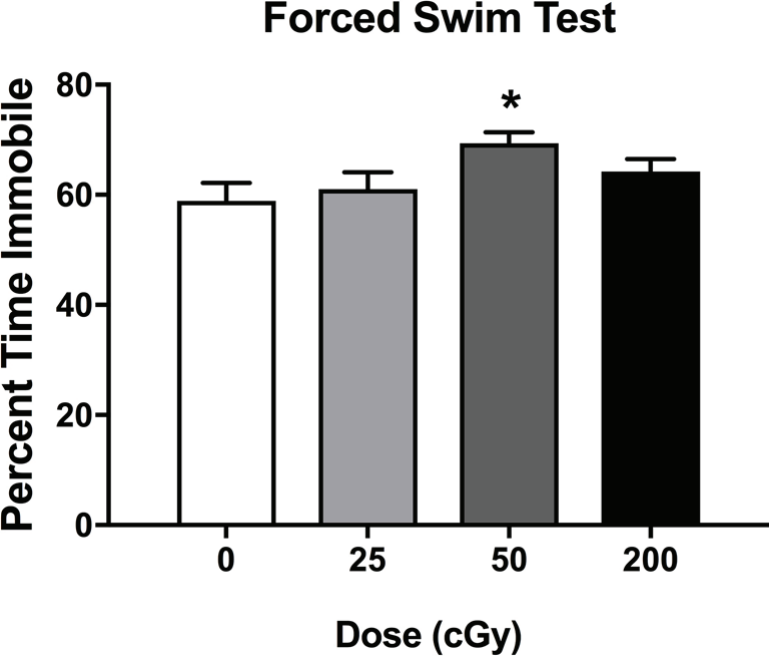
Depressive behavioral performance of sham-irradiated mice and mice irradiated with mixed beams in the forced swim test. There was a trend toward an effect of irradiation on time spent immobile during the forced swim test (*F*_3,78_ = 0.567; *p* = 0.057). Mice irradiated with 50 cGy spent more time immobile than sham-irradiated mice (*p* = 0.026, Dunnett’s test).

Effects of rapid, sequential three-beam irradiation on depressive behavior were assessed in the forced swim test. Mice irradiated with 50 cGy spent more time immobile than sham-irradiated mice (*p* = 0.026, Dunnett’s test), although there was not a significant effect of irradiation when all doses were considered together. (Figure 3, *F*_3,78_ = 0.567; *p* = 0.057). There was no effect of sex on performance in the forced swim test (*F*_1,78_ = 0.285; *p* = 0.595).

### Fear Conditioning

**Figure 4:**
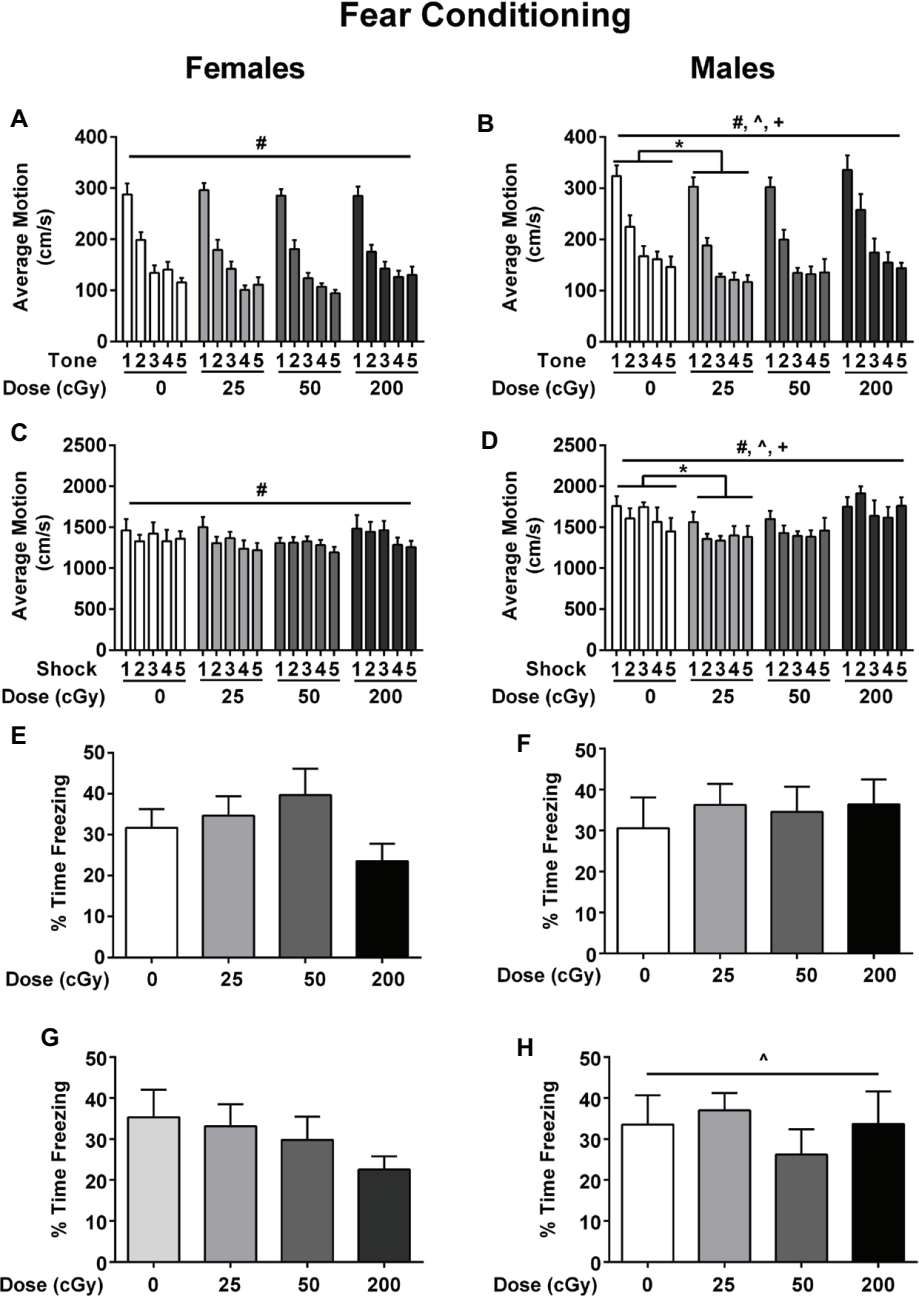
Performance of female **(A)** and male **(B)** sham-irradiated mice and mice irradiated with mixed beams in the fear conditioning test. **(A/B)** Males moved more than females during the tones, especially in the 200 cGy dose group (*F*_1,3_ = 11.025; *p* = 0.001). There was also an effect of irradiation (*F*_3,75_ = 3.040; *p* = 0.034). Male mice irradiated with 25 cGy moved less than sham-irradiated male mice during the tones (*p* = 0.0075). **(C/D)** There was an effect of sex (*F*_1,75_ = 17.407; *p* < 0.001) during the shocks; males moved more than females during the shocks. There was also an effect of irradiation (*F*_3,75_ = 1.237; *p* = 0.017); male mice irradiated with 25 cGy moved less than sham-irradiated male mice (*p* = 0.0305). **(E/F)** There were no effects of sex (*F*_1,75_ = 0.245, *p* = 0.622), irradiation (*F*_3,75_ = 0.678, *p* = 0.568), or a sex × irradiation interaction (*F*_3,75_ = 0.777, *p* = 0.511) for contextual fear memory. **(G/H)** Females moved more than males (*F*_1,75_ = 5.092; *p* = 0.027, ANOVA; by tone *F*_1,75_ = 5.529; *p* = 0.021, repeated measures ANOVA) but there were no effects of irradiation (*F*_3,75_ = 1.123, *p* = 0.345), sex *F*_1,75_ = 0.627, *p* = 0.431), or radiation × sex interaction (*F*_1,75_ = 0.427, *p* = 0.734) for cued fear memory.

Emotional learning and memory were assessed in the contextual and cued fear conditioning tests. On the training day, average motion (cm/s) was assessed during a 90-s baseline period, during each of the five tones, during each of the five shocks, and during each of the five inter-stimulus intervals (ISIs). During the baseline period, there were no effects of irradiation (*F*_1,3_ = 0.373; *p* = 0.772), sex (*F*_1,1_ = 0.153; *p* = 0.697), or a sex × irradiation interaction (*F*_1,3_ = 1.175; *p* = 0.325). Analysis of average motion during the tones, using a repeated measures ANOVA, showed that there was an effect of sex (*F*_1,3_ = 11.025; *p* = 0.001) with males (Figure 4B) moving more on average than females (Figure 4A), especially in the 200 cGy dose group. There was also an effect of irradiation during the tones (*F*_3,75_ = 3.040; *p* = 0.034); male mice irradiated with 25 cGy moved less than sham-irradiated male mice (Figure 4B, *p* = 0.0075). Similarly, there were also significant main effects of sex (*F*_1,75_ = 17.407; *p* < 0.001) during the shocks, with males (Figure 4B) moving more than females (Figure 4A). There was also an effect of irradiation (*F*_3,75_ = 1.237; *p* = 0.017); during the shocks, male mice irradiated with 25 cGy moved less than sham-irradiated male mice (*p* = 0.0305, Figures 4C,D). There were no significant effects of radiation or sex on average freezing or motion during the ISI periods.

Next, contextual fear memory was assessed (females: Figure 4E; males: Figure 4F). There were no effects of sex (*F_1,75_* = 0.245, *p* = 0.622), irradiation (*F_3,75_* = 0.678, *p* = 0.568), or a sex × irradiation interaction (*F_3,75_* = 0.777, *p* = 0.511) for the percent time freezing over the 5 min of the trial. Although females in the 200 cGy group appeared to display more freezing behavior than sham-irradiated female mice (Figure 4E), this did not reach statistical significance.

Next, cued fear memory was assessed (Figures 4G,H). All groups froze more during the tones than during the period prior to the tone (pre-tone period: *F*_1,75_ = 251.708; *p* < 0.001, ANOVA). Average motion and percent time freezing were then analyzed for cued associative memory. Females moved more than males (total motion *F*_1,75_ = 5.092; *p* = 0.027, ANOVA; by tone *F*_1,75_ = 5.529; *p* = 0.021, repeated measures ANOVA). There were no effects of irradiation (*F_3,75_* = 1.123, *p* = 0.345), sex (*F_1,75_* = 0.627, *p* = 0.431), or radiation x sex interaction (*F_3,75_* = 0.427, *p* = 0.734) for cued fear memory (Figures 4G,H).

### Passive Avoidance

Emotional learning and memory were also assessed in the passive avoidance test. On day 1 of the passive avoidance test (training), females showed a significantly shorter latency to cross to the dark compartment than males (*F*_1,75_ = 4.689; *p* = 0.034, ANOVA, not shown). This may indicate a higher level of anxiety in the tested female than male mice. There was no effect of rapid, sequential three-beam irradiation on latency to enter the dark compartment on day 1. On day 2, latency to enter the dark compartment was assessed. Analysis showed that females were quicker to cross to the dark compartment than males (*F*_1,75_ = 8.362; *p* = 0.005, ANOVA, not shown). There was no effect of irradiation on latency to enter the dark compartment on day 2 (*F*_1,75_ = 0.95; *p* = 0.421, ANOVA), nor was there a sex × radiation interaction (*F*_1,75_ = 0.2; *p* = 0.896, ANOVA, not shown).

### Cortical BDNF, CD68, and MAP-2 Levels

**Figure 5:**
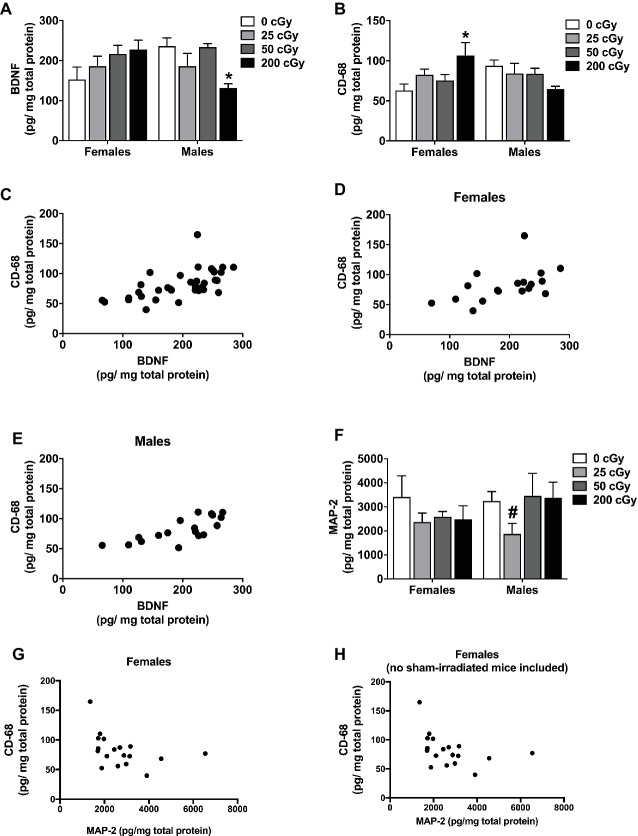
Protein quantification of cortical BDNF, CD68, and MAP-2. **(A)** Cortical BDNF levels of sham-irradiated mice and mice irradiated with mixed beams. There was a sex × radiation interaction (*F*_3,35_  = 4.772, *p* = 0.008). In male mice, there was an effect of irradiation in male mice **(A)** (*F*_3,17_ = 5.040, *p* = 0.014). BDNF levels in male mice irradiated with 200 cGy were lower than those in sham-irradiated male mice (*p* = 0.014, Dunnett’s test). No significant effects were seen in female mice. **(B)** Cortical CD68 levels of sham-irradiated mice and mice irradiated with mixed beams. There was a sex × radiation interaction (*F*_3,17_ = 5.040, *p* = 0.009). In female mice, there was a trend toward an effect of irradiation on CD68 levels (*p* = 0.068). CD68 levels were higher in mice irradiated with 200 cGy than sham-irradiated mice (*p* = 0.030, Dunnett’s test). No significant effects were seen in male mice. **(C)** There was a positive correlation between BDNF and CD68 when the female and male data were analyzed together (*r* = 0.6762, *p* < 0.0001, Spearman correlation). **(D)** There also was a positive correlation between BDNF and CD68 levels when the female (*r* = 0.5769, *p* = 0.0122, Spearman correlation) and male (*r* = 0.7688, *p* = 0.0002, Spearman correlation) data were analyzed separately. **(E)** There was a positive correlation between BDNF and CD68 levels when the female and male data were analyzed together (*r* = 0.6015, *p* = 0.0001, Pearson correlation). **(F)** Cortical levels of MAP-2 in sham-irradiated and mice irradiated with mixed beams show no difference due to sex (*F*_1,28_ = 0.361, *p* = 0.553) or dose (*F*_3,28_ = 1.253, *p* = 0.309). Male mice show a trending difference between sham-irradiated mice and mice irradiated with 25 cGy (*p* = 0.057, *t*-test). **(G)** In females, but not in males, there was a negative correlation between MAP-2 and CD68 (*r* = −0.473, *p* = 0.0226, Spearman correlation). **(H)** This negative correlation remained when data of sham-irradiated female mice were removed (*r* = −0.5191, *p* = 0.0273, Spearman correlation).

Cortical tissues of the mice were used for analyses of BDNF, CD68, and MAP-2 levels as a function of radiation dose. For BDNF, there was a sex × radiation interaction (*F*_3,35_ = 4.772, *p* = 0.008). Therefore, the female and male data were analyzed separately. In females, there was no significant effect of irradiation (Figure 5A, *p* = 0.198). However, there was a significant effect of irradiation in male mice (Figure 5A, *F*_3,17_ = 5.040, *p* = 0.014). BDNF levels in male mice irradiated with 200 cGy were lower than in sham-irradiated male mice (*p* = 0.0142, Dunnett’s test).

For cortical CD68 levels, there was also a sex × radiation interaction (*F*_3,17_ = 5.040, *p* = 0.009). CD68 levels were higher in female mice irradiated with 200 cGy than in sham-irradiated females (Figure 5B, *p* = 0.030, Dunnett’s test); however, there was not a significant effect of irradiation overall (Figure 5B, *p* = 0.068). There was no significant effect of irradiation on CD68 levels in males (Figure 5B, *F*_3,17_ = 5.040, *p* = 0.219).

We also analyzed the relationship between BDNF and CD68 levels in individual mice. There was a significant positive correlation between BDNF and CD68 levels when the female and male data were analyzed together (Figure 5C, *r* = 0.6762, *p* < 0.0001, Spearman correlation) and when the female and male data were analyzed separately (Figure 5D, females: *r* = 0.5769, *p* = 0.0122, Spearman correlation; Figure 5E, males: *r* = 0.7688, *p* = 0.0002, Spearman correlation). The significant positive correlation between BDNF and CD68 levels was also seen when the data from the mice that received sham irradiation were removed from the analysis (*r* = 0.7023, *p* = 0.0072, Pearson correlation), suggesting that differences in BDNF and CD68 levels between sham-irradiated and irradiated mice are not required to reveal this relationship. In addition, this relationship seems to depend on radiation exposure, as no relationship between cortical BDNF and CD68 levels was seen in sham-irradiated B6D2F1 mice (*r* = 0.06, *p* = 0.8).

Although there are no significant differences due to either sex (*F*_1,28_ = 0.361, *p* = 0.553) or dose (*F*_3,28_ = 1.253, *p* = 0.309) in MAP-2 levels (Figure 5F), we also assessed the relationship between MAP-2 and BDNF and MAP-2 and CD68. In females only, there was a negative correlation between MAP-2 and CD68 (*r* = −0.473, *p* = 0.0226, Spearman correlation, Figure 5G). This negative correlation remained when data of sham-irradiated female mice were removed (*r* = −0.5191, *p* = 0.0273, Spearman correlation, Figure 5H).

### Microbiome

**Figure 6:**
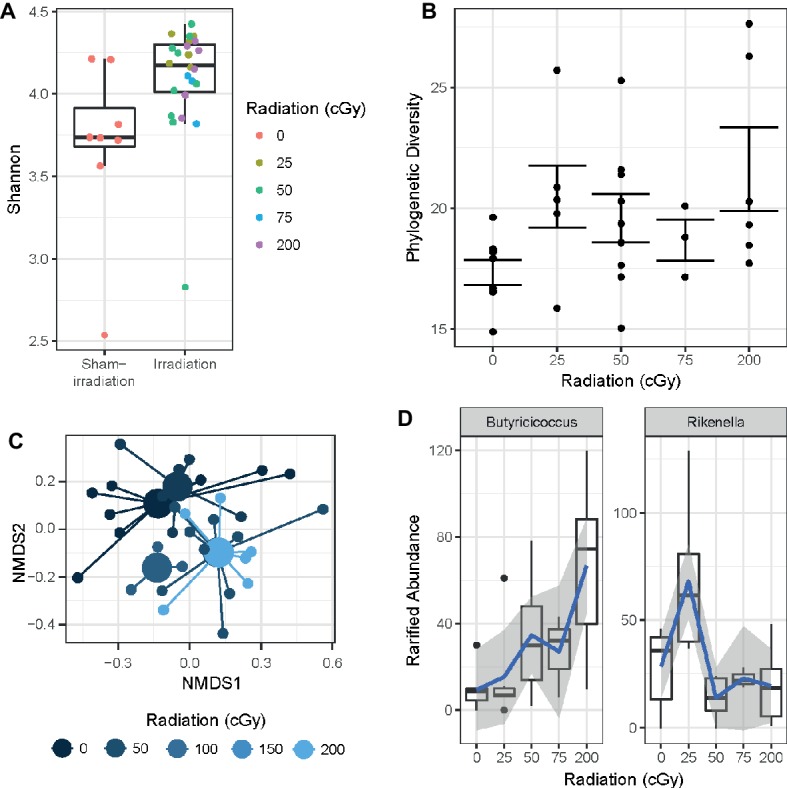
**(A)** Sequential beam irradiation increased alpha-diversity of the gut microbiome in a dose-dependent fashion as measured by the Shannon entropy statistic (Wilcoxon test *p* < 0.005). **(B)** The phylogenetic diversity of the gut microbiome increased as a function of dose (linear model slope: 0.17, *p* = 0.035). **(C)** Composition of the gut microbiome is significantly associated with dose based on the Canberra distance metric (*p* = 0.007). **(D)** Several taxa vary in terms of the relative abundance in the gut as a function radiation exposure, including *Butyricicoccus* (left) and *Lachnospiraceae* (right) (Kruskal-Wallis *fdr* < 0.2). Blue lines represent a loess fit through the distribution of observations, where gray areas represent the standard error.

Bacterial DNA from mouse fecal pellets was extracted, and the V4 subregion of the 16S rDNA gene was amplified and sequenced to determine the diversity and composition of the intestinal microbiome. Exposure to rapidly delivered, sequential three-beam irradiation increased the alpha-diversity of the gut microbiome relative to unexposed individuals as measured by the Shannon entropy statistic (Wilcoxon test *p* < 0.005; Figure 6A), a metric of community evenness. Moreover, increasing the dose of sequential three-beam radiation increased the phylogenetic diversity of the gut microbiome as measured by a linear model (slope: 0.17, *p* = 0.035; Figure 6B). A PERMANOVA test found that composition of the gut microbiome, as measured by the Canberra distance metric, significantly associates with the dose of the sequential three-beam irradiation to which the host is exposed (*p* = 0.007; Figure 6C). Beta-diversity (measure based on comparison of samples to each other) was not associated with any of the other covariates measured as part of the study, though this observation may be driven by sample size. Non-parametric tests of association as measured by Kendall’s tau revealed several genus-level phylotypes whose abundance in the gut varies in association with radiation dose (fdr < 0.2): *Alistipes*, *Butyricicoccus, Enterorhabdus, Lachnospiraceae, Marvinbryantia, Rikenella,* and *Ruminiclostridium*. As shown in Figure 6D, these taxa do not always manifest monotonic relationships with the radiation dose. For example, while *Butyricicoccus* positively increases as a function of dose, taxa like *Rikenella* manifest a peak relative abundance after exposure to 25 cGy.

Having observed changes in microbiome composition as a result of space radiation exposure, we assessed whether there were significant microbiome associations with all behavioral and cognitive measures. There were no significant associations between the microbiome diversity or microbiome composition and behavioral or cognitive measures.

## Discussion

This study shows that rapidly delivered, sequential three-beam radiation can have detrimental effects on the mouse brain. These effects appear only for certain endpoints, often at discrete doses, and the effects can differ between males and females. In a few cases, there are common responses in both sexes. For example, exposure to either 50 or 200 cGy (total dose) impairs object recognition. The pattern seen would suggest that the threshold for cognitive injury might be between 25 and 50 cGy for this endpoint. The sensitivity of the object recognition test to detect detrimental effects of rapid, sequential three-beam irradiation in B6D2F1 mice on cognitive performance is consistent with data in B6D2F1 mice following ^4^He ion irradiation (250 MeV/n, 42 cGy) (Raber et al., 2018). In addition, in C57BL/6J mice, object recognition is also sensitive to detect detrimental effects of ^56^Fe ions (Impey et al., 2016a,b), ^28^Si ion (Raber et al., 2014a,b), or proton (Impey et al., 2017) irradiation on object recognition at 6 months of age.

As depressive-like symptoms were reported to be altered in astronauts (Slack et al., 2016) and can affect cognitive performance, we assessed depressive-like behaviors in this study. Depressive behavior in the forced swim test and activity in the home cage during the active period were only altered following three-beam irradiation at a dose of 50 cGy. Thus, alterations in depressive behavior and home cage activity are not required for revealing impairments in cognitive function following exposure to a complex mixture of light and heavier ion beams. However, we cannot exclude that in mice irradiated with 50 cGy, these behavioral alterations contributed to the cognitive injury seen in the mice.

In contrast to object recognition, no effects of irradiation were seen in the present assessments of contextual or cued fear memory. Notably, other individual ions that are components of space irradiation, including ^56^Fe ions (Villasana et al., 2010a,b; Raber et al., 2013a,b), or ^28^Si ions (Raber et al., 2014a,b; Raber et al., 2015a,b,c; Whoolery et al., 2017) affected contextual fear memory, while ^16^O ion irradiation affected cued fear memory (Raber et al., 2015a,b,c), as did ^28^Si in one study (Whoolery et al., 2017), while ^40^Ca ion exposure affected performance during fear conditioning training without affecting contextual fear memory (Raber et al., 2016). Thus, different components of the space environment affect distinct behavioral and cognitive measures. However, it is conceivable that these distinct behavioral and cognitive effects might be part of a temporal cascade of CNS dysregulation that contribute to an increased allostatic load and ultimately to enhanced CNS risk (Juster et al., 2010). In any event, based on what is seen with a single ion, it is hard to predict how combined exposures including any given ion might affect brain function. We recognize that in addition to space radiation, astronauts will also be exposed to microgravity and other stressors that might modulate the radiation response (Pecaut et al., 2000; Ray et al., 2001; Bederman et al., 2013; Bellone et al., 2016; Chowdhury et al., 2016).

Neuroinflammation, including microglial activation, has been identified as a mediator of behavioral alterations and cognitive impairments in many neurological conditions, including Alzheimer’s disease, major depressive disorder, and injury from cerebral ischemia (Akiyama et al., 2000; Moravan et al., 2011; Fuster-Matanzo et al., 2013). Neuroinflammation has also been associated with depressive behavior (Brites and Fernandes, 2015). Recent research supports the role of neuroinflammation in mediating the neurological dysfunction seen with cancer and cancer treatment (McGinnis et al., 2017; McGinnis and Raber, 2017). Neuroinflammation has also been associated with cognitive injury in animal models following irradiation (Moravan et al., 2011; Belarbi et al., 2013; Raber et al., 2013a,b; Raber et al., 2015a,b,c). However, the sex-dependent alterations in CD68 levels seen in the present study with sequential three-beam radiation highlight the complex relationship between immune activation and brain function. In females, radiation-induced cognitive injury was associated with increased CD68 levels, while in males, radiation-induced cognitive injury was associated with reduced CD68 levels. These sex-dependent effects of rapid, sequential three-beam irradiation on cortical BDNF and CD68 levels suggest that distinct pathways might be involved in cognitive injury in females and males (Gray, 1971; Beatty et al., 1973; Gray and Lalljee, 1974; Patsch et al., 1980; Kemble and Enger, 1984; Lipa and Kavaliers, 1990; Akinci and Johnston, 1993; Maren et al., 1994; Klein and Flanagan, 2016). Increased efforts are warranted to use unbiased approaches to determine altered pathways in females and males that might underlie the potential effects of the complex radiation fields in deep space for each sex.

Alterations in the gut microbiome following space radiation might be important for effects on the brain (Ritchie, 2015; Cervantes and Hong, 2016; Turroni et al., 2017). The sequential three ion beam exposures used in the present study increased the alpha-diversity of the gut microbiome and significantly altered the composition of the gut microbiome. While the potential effects of space radiation on the gut microbiome has been recognized as a challenge to long-duration space missions (Voorhies and Lorenzi, 2016), relatively little is known about the direct impact of such radiation on the gut microbiome. Most work thus far has only assessed a limited set of cultured gut microbes, though more comprehensive methods are being applied as part of the ongoing Astronaut Microbiome project (Voorhies and Lorenzi, 2016). In one study, the authors exposed mice to continuous low doses (0–1 Gy/day) of high linear energy transfer radiation (Casero et al., 2017). Despite differences in study design, including variation in the types of irradiation, the length of exposure, and the dose, our findings are generally consistent with that study. For example, those authors showed alterations to gut microbiome composition at low exposure doses, and we observe a similar dose-dependence of the three-beam irradiation’s effect on gut microbiome composition. Those authors also observed dependences between radiation dose and the relative abundance of specific gut microbes, but noted that these changes were not monotonic over the range of doses. We also find that these types of patterns manifest at higher doses, at least for the genera *Marvinbryantia*, *Enterorhabdus*, *Rikenella*, and *Ruminiclostridium*. However, we also find that *Butyricicoccus* and *Lachnospiraceae* UCG-001 monotonically increase with exposure, which is notable given that prior work links these butyrate-producing genera to protection from intestinal inflammation (Eeckhaut et al., 2013). Our finding suggests that perturbation of the gut microbiome in the irradiated mice may not necessarily have an adverse health consequence. The effects on the gut microbiome seen are not necessarily a result of just radiation exposure. We recognize that microbiota vary a lot across body habitats (Costello et al., 2009) and that a combination of the environments the mice were in at BNL, OHSU, and during transport, behavioral testing, and radiation exposure might all have contributed to the effects seen on the gut microbiome.

Collectively, these studies underscore the sensitivity of the gut microbiome’s composition to components of the space radiation environment and justify future investigations of the health impacts of space irradiation-induced alterations to the gut microbiome. Recently, we reported significant associations between microbiome alpha-diversity and sensorimotor performance, as well as microbiome composition and fear learning in a MPTP Parkinson’s disease mouse model of brain injury, (Torres et al., 2018). We did not detect such associations in the current study. However, recognizing the limitations of only using the current three-beam sequential exposure paradigm to model the space environment astronauts are exposed to during missions and the power limitations of the current study, future analyses are warranted to determine whether following other sequential mixed beam exposures, alterations in the gut microbiome are associated or even correlated with specific behavioral and/or cognitive measures. In addition, future efforts are warranted to assess how radiation affects the gut microbiome, including whether radiation directly impacts the microbiome and whether radiation-dependent effects on the intestinal epithelium, gut immune system, or gut-brain axis (Carabotti et al., 2015) contribute to these impacts.

In summary, sequential proton, ^16^O ion, and ^28^Si ion irradiation affects home cage activity, depressive behavior in the forced swim test, object recognition, cortical levels of BDNF, and CD68, and the diversity and composition of the gut microbiome. Different outcome measures show distinct dose–response relationships and some, but not all, of the affected outcome measures are sex-dependent. As astronauts are exposed to a mixture of distinct charged particles during missions, increased efforts are warranted to assess the effects of complex, sequential beam exposures on the brain and to define altered pathways in and outside the brain that might mediate these effects.

## Author Contributions

JR, AK, and MT conceived and designed the work. JY, ET, NK, KS, and TS performed the experiments. JR, JY, ET, KS, and TS analyzed the data. JR, AK, TS, and MT wrote the paper.

### Conflict of Interest Statement

The authors declare that the research was conducted in the absence of any commercial or financial relationships that could be construed as a potential conflict of interest.
